# The use of Fat-Augmented Latissimus Dorsi (FALD) flap for male Poland Syndrome correction: a case report

**DOI:** 10.1080/23320885.2022.2117701

**Published:** 2022-09-08

**Authors:** Fabio Santanelli di Pompeo, Michail Sorotos, Guido Paolini, Gennaro D’Orsi, Guido Firmani

**Affiliations:** a Department NESMOS – Sant’Andrea Hospital, Faculty of Medicine and Psychology, Sapienza University of Rome, Rome, Italy; b Chair of Plastic Surgery, Faculty of Medicine and Psychology, Sapienza University of Rome – Sant’Andrea Hospital, Rome, Italy

**Keywords:** Poland Syndrome, FALD flap, latissimus dorsi flap, autologous fat transfer, lipofilling, congenital disease

## Abstract

We present a 23-year-old male patient with severe PS, characterized by marked left thoracic wall deformity. Reconstruction was performed using the Fat-Augmented Latissimus Dorsi flap, which was fixed to the chest wall hollowing corresponding to where the pectoralis major muscle was missing. Patient was satisfied with final aesthetic and functional result.

## Introduction

Poland Syndrome (PS) is a sporadic congenital disease with a wide spectrum of presentations [1,2]. Typical features include the absence of the sternum-costal head of the pectoralis major (PM) muscle, along with glandular and subcutaneous tissue hypoplasia and thin overlying skin. Sometimes the nipple-areola complex (NAC) appears underdeveloped or missing. Clinical presentation may include skeletal deformities, costal cartilage and rib defects, ipsilateral upper limb malformations or elevation and rotation of the scapula, also known as Sprengel deformity [3]. The causes for PS are unknown, although a disruption of the embryonic blood supply to the subclavian arteries seems to be the most accepted hypothesis [4]. The prevalence at birth of this syndrome is of about 1–3 in 100.000 individuals, with male predominance at a ratio of 2:1–3:1 [5].

In male patients, the lack of the PM muscle causes various degrees of chest asymmetry with the absence of an anterior axillary fold, often worsened by concomitant rib cage anomalies. These malformations of the chest wall usually lead to discomfort, which can sometimes force patients into social withdrawal or cause noticeable psychological trauma.

Several methods for male PS correction have been reported in literature, including tissue expanders, custom silicone implants, autologous fat transfer (AFT) and a variety of flap options [6–9].

The aim of this paper is to extend the range of applications for the Fat-Augmented Latissimus Dorsi (FALD) flap, a procedure originally introduced in 2014 for post-mastectomy breast reconstructions [10], to correct a severe case of male PS, thereby extending the range of autologous-based options now available in the plastic surgeon’s armamentarium.

## Case report

**Figure 1.**
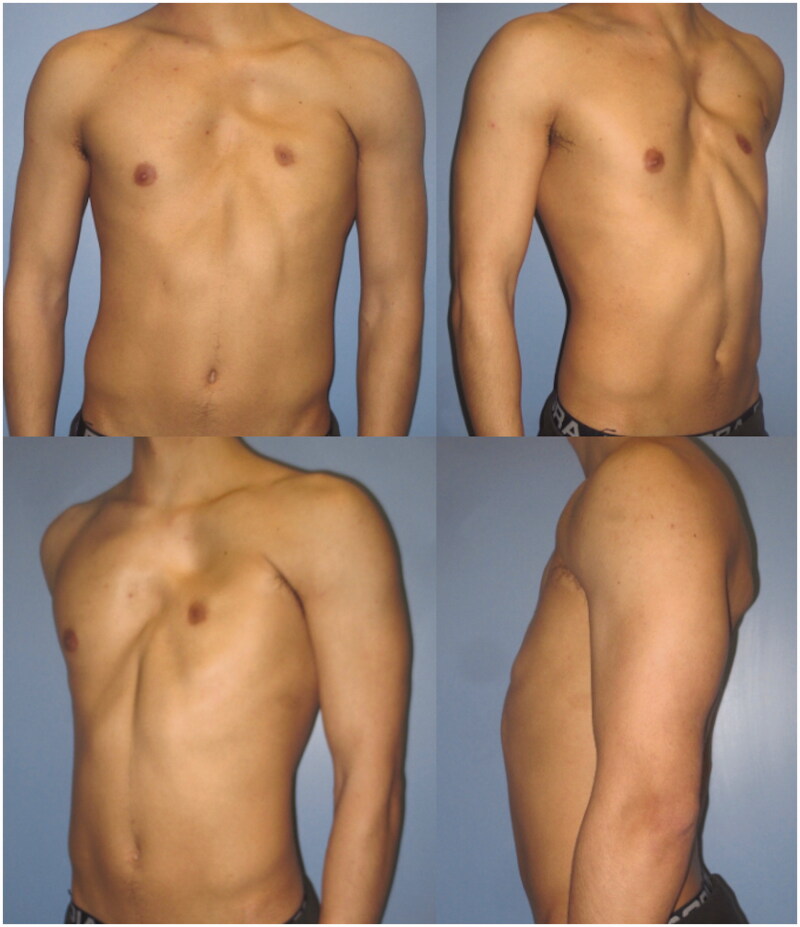
Pre-operative photographs of the 23-year-old patient, in the frontal (upper left), right oblique (upper right), left oblique (lower left) and lateral (lower right) projections.

A 23-year-old male patient came to our attention with noticeable anterior thoracic wall asymmetry and left rib cage deformity, suggestive for PS. The patient presented a severe infra-clavicular hollowing on the left thoracic wall caused by lack of soft tissues along with a deformity of the costal cartilage ribs. This was classified as grade 3 severity according to Foucras classification. The left NAC appeared displaced toward the axilla, where the left anterior axillary pillar was missing (Figure 1). No limb deformity nor any alteration in the contralateral chest wall were observed. The ipsilateral LD muscle showed no morphological alteration during clinical examination. A chest Magnetic Resonance Imaging (MRI) scan was performed prior to surgery, showing agenesis of the left PM muscle belly, hypoplasia of the left mammary gland and a depression of the second, third, fourth and fifth chondro-costal junctions [11].

The reconstructive process consisted of a pedicled FALD flap. The preoperative markings were performed the day prior to surgery, with the patient in the upright position. The largest possible transverse skin paddle was drawn on the back (19.0 cm × 8.0 cm) using the pinch test. The major axis of the skin paddle was drawn slightly tilted compared to an imaginary transverse horizontal line, which allowed an easy closure of the donor site. The surgery started with the patient in right lateral decubitus position and with the left upper limb suspended at a right angle, to provide adequate axillary access. The skin paddle was first de-epithelialized, then its edges were incised perpendicularly through the Scarpa’s fascia and down to the muscle fascia. The LD muscle was then harvested in its entirety, dissecting proximally up to the insertion tendon on to the intertubercular groove of the humerus, keeping the thoracolumbar fascia intact on the back. The thoracodorsal pedicle was identified from below, isolated and dissected proximally, until reaching the required length for tension-free flap transposition. The thoracodorsal nerve was not sectioned to avoid late muscle atrophy, in order to perform a functional reconstruction of the left thoracic wall. A suction drain was placed at the donor-site, which was closed in two layers. An incision was performed on the left thoracic wall to provide an adequate view of the recipient site and avoid pneumothorax or pericardial injury. The recipient area was prepared, extending dissection from the anterior axillary line to the left parasternal line and from the manubrium to the xiphisternal line.

**Figure 2.**
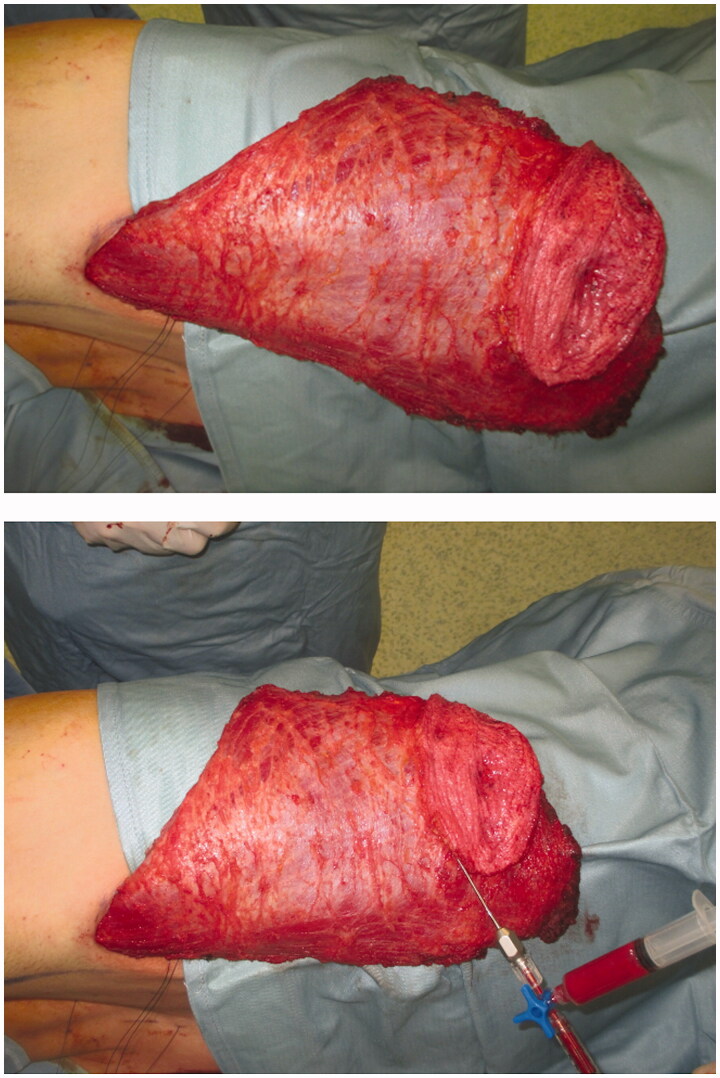
Fully harvest FALD flap (above) and immediate AFT into the dermo-adipose skin paddle (below), following our previously described “Simplified lipostructure technical note”.

Fat was harvested simultaneously from the left trochanteric region using 2.3 mm cannulas, collecting 120 cc of fat in total. Harvested fat was processed using Coleman’s technique, yielding 80 cc of injectable fat, which was later distributed into the superficial and deep adipose layers of the LD dermo-adipose paddle as well as under the LD muscle fascia, cranially to the paddle. We used 1-ml syringes for fat tissue injection, in accordance with our previously described [12] ‘simplified lipostructure technical note’ (Figure 2) [13].

After shifting the patient to the supine position, the flap was rotated through a subcutaneous tunnel to the anterior chest wall. Subsequently, to replace the anatomy of the missing PM muscle, the mid shaft of the LD was sutured to the periosteum of the costal bone to restore the pectoral skinfold and the anterior axillary pillar, while the distal shaft was folded over to position the fat-augmented dermo-adipose paddle where the chest hollowing was greatest. Finally, a suction drain was placed as we proceeded with layered skin closure.

The operative time was 4.00 h. The patient had an uneventful postoperative course and was discharged on the fourth postoperative day. No seroma or other complications occurred at donor site and no fat transfer–related complications were observed neither in the recipient nor in the donor site. After the FALD flap procedure, symmetry was further improved with one delayed AFT session at 3 months. This second procedure was performed in an outpatient setting under local anaesthesia and had an uneventful post-operative course as well.

## Discussion

Correction of severe chest deformities in male PS can represent an important challenge for plastic surgeons. Implant-based reconstructions are the most commonly used techniques, where custom-made implants are used to fill-in the hollowed areas on a case-by-case basis [14,15]. Nevertheless, the high likelihood of developing implant-related complications leads us to stray away from using prosthetic approaches [16]. These complications include but are not limited to exposure, extrusion, rupture, infection, capsular contracture and the widely accepted Breast Implant Associated-Anaplastic Large Cell Lymphoma (BIA-ALCL) [17–20]. Chavoin et al. in 2018 reported a case series of 68 consecutive patients treated with three-dimensional computer-aided design silicone implant [21]. They reported a periprosthetic seroma rate of 20.6% that required puncture before resolving spontaneously. In addition, these reconstructions are common in young patients, thus requiring several surgical revisions for implant size and shape replacement for the rest of their life [22–24]. For all of these reasons, we favour autologous-based approaches to treat chest deformities in PS, especially for male patients [25]. Amoroso et al. first described the use of LD flap for correction of PS deformities in 1981 [26]. Extended LD flap harvest can cause increased donor site morbidity, including seromas, wound dehiscence and lumbar hernias, with a rate as high as 38.7% [27,28]. Nevertheless, we found our donor site seroma rate to be 0% in our previously published research based on a case series of 25 breast reconstructions [10]. We found this rate to still be remarkably low (0.68%) in a much larger case series of 148 breast reconstructions using FALD, which is currently still in press [29]. This is likely due to considerable technical differences. Those include closing the donor defect under high tension to reduce the dead space to a minimum, and divert most tension to the deep Scarpa’s fascia plane using 2-0 absorbable multifilament interrupted stitches. Additionally, we use 3-0 absorbable multifilament stiches for superficial subdermal plane and 3-0 absorbable antibiotic-laced multifilament threads for running intradermal sutures. A vacuum drain is left in place at the very lateral end of the wound.

Seyfer et al. [30] described several chest wall reconstruction procedures in 22 male patients with PS, among which 12 cases underwent LD muscle transfer. Yesilada et al. [31] discuss 30 cases of congenital, developmental and acquired breast asymmetries in female patients, including PS, treated with tissue expanders with or without muscle flaps, accompanied by delayed AFT sessions which were used for correcting hollowed areas, and not for enhancing the volume of the skin paddle. Limitation to these procedures include the lack of volume, especially when the chest wall malformation creates large depressions, i.e. grade 2 and 3 in the Foucras severity classification [11]. Other authors discuss the feasibility of LD flap for PS in a female patient, which was later followed by delayed AFT [25]. These are considerably different from the FALD flap, which can guarantee greater volumes by simultaneously enriching the skin paddle with AFT,. This has the added benefit of keeping the reconstruction fully autologous, thus avoiding prosthetic devices altogether. Other autologous alternatives described in literature include free flaps such as the transverse musculo-cutaneous gracilis flap or the laparoscopically harvested omental flap [32,33]. Both these options also provide autologous tissues. However, they are more demanding for the surgeons as well as the patients, requiring longer operating times and microsurgery skills. As microsurgery has reached a level of popularity where it has now been democratized, in an era of constraints we must ensure that allocated resources for operating rooms are used responsibly [34]. Therefore, when similar results can be achieved, surgeons should favour the least complex and shortest possible alternative in the ladder of surgical options.

When discussing LD flap-based procedures, it is worthwhile mentioning how other surgeons have established minimally invasive procedures aimed at avoiding the creation of a back scar. Those include endoscopic or robotic approaches, which have been popularized for some breast or head and neck reconstruction cases [35–37]. Compared to open techniques, they provide advantageous aesthetic results of the donor site, however they are not practiced in all centers due to the increase in surgery costs, the steep learning curve and the rare potential for insufflation-related morbidity.

Replacement of the PM muscle morphology and restoration of its function are key steps in male PS correction. In our case, the thoracodorsal nerve supporting the LD muscle was kept intact to perform a functional muscle transfer, preserving the humerus adduction capacity to the thorax. Furthermore, the LD muscle was located in correspondence of the absent PM, in order to substitute it not only in shape and morphology but also in function. We tightly sutured the LD muscle to the costal ribs periosteum, to mimic the new anterior axillary pillar with a good definition and ensuring muscular function similar to the PM muscle. Finally, adequate filling of the hollowing in the infra-clavicular area was achieved using the fat enriched dermo-adipose paddle, which was fixed to the area sinking the most.

**Figure 3.**
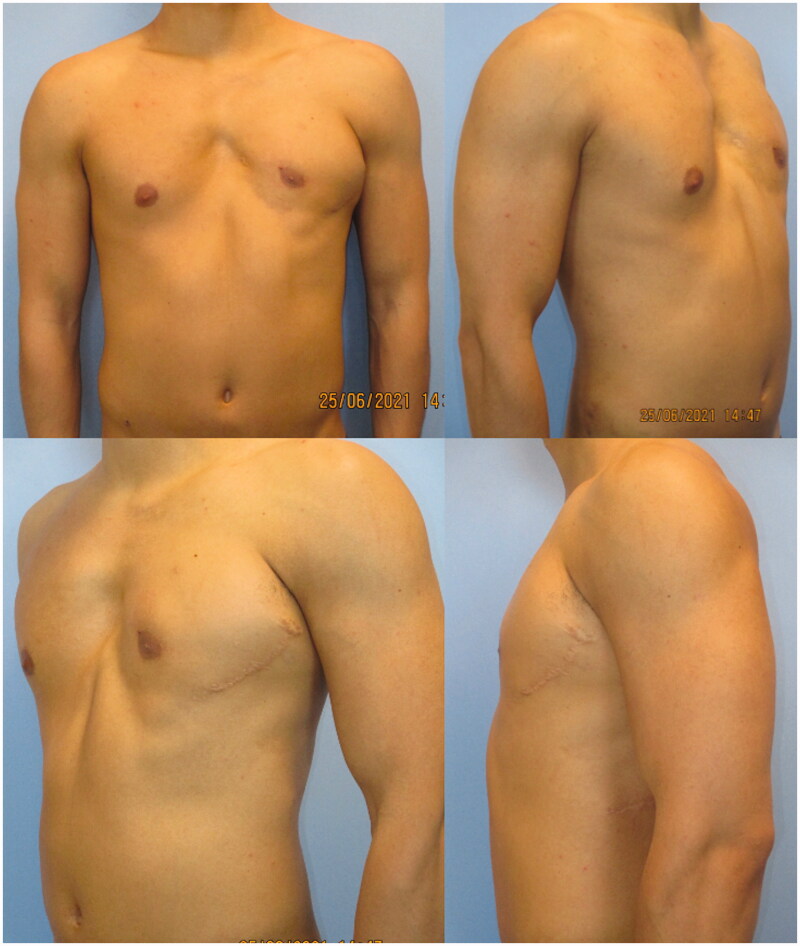
Post-operative result at 18-month follow-up, in the frontal (upper left), right oblique (upper right), left oblique (lower left), and lateral (lower right).

**Figure 4.**
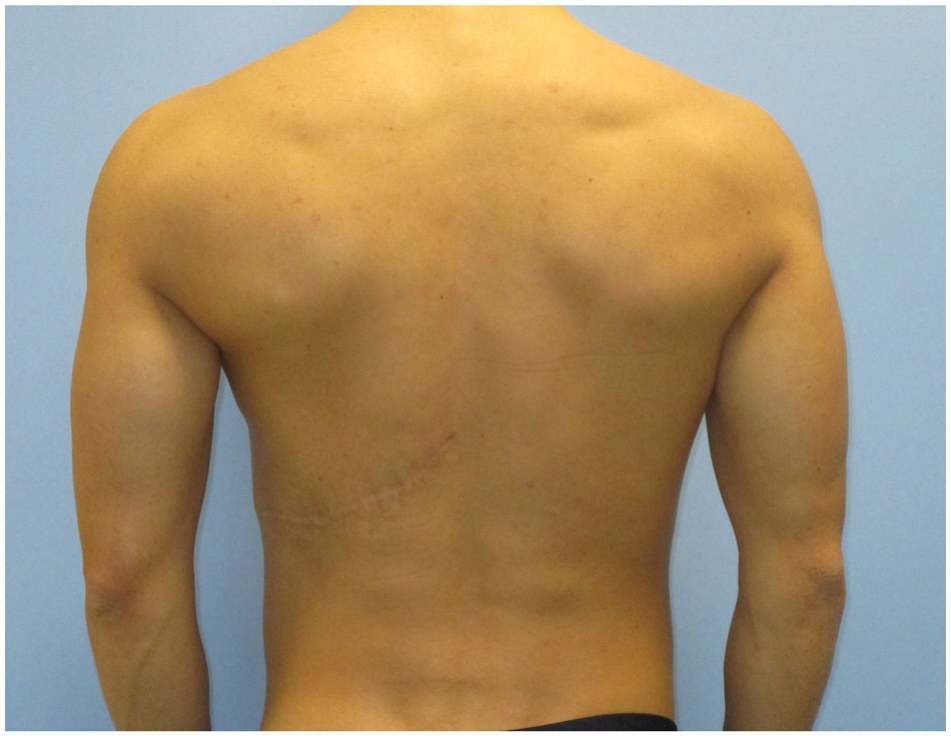
Post-operative result at 18-month follow-up in the posterior projection.

Despite the undercorrection of the lower inner-quadrant defect, which could have been solved with an additional fat grafting session, the patient was satisfied with the final aesthetic and functional result, and refused additional procedures. He described an increase in his self-confidence and in the appreciation of his physical appearance (Figures 3 and 4). His arm functionality was unaffected and he also reports a resumption of his hypertrophy-centered training regimen at the gym, at 12 months from the procedure. To the best of our knowledge, this is the first report described in literature of a correction of chest wall deformity in PS using FALD flap.

## Conclusion

Correcting male PS deformities remains a challenge for plastic surgeons. We increased the catalogue of available autologous-based options by extending the indications of the FALD flap, originally used for breast reconstruction alone. The possibility of adding fat into the dermo-adipose paddle above the muscle guarantees excellent results to correct chest wall deformities, thus avoiding the use of customized silicone implants and all their related sequelae. At the same time, functional LD muscle transfer simultaneously replaces the PM muscle function and morphology. In our case, totally autologous PS correction was achieved using the FALD flap and one additional AFT session, obtaining optimal aesthetic results and satisfactory symmetry.
